# Targeting Enclysis in Liver Autoimmunity, Transplantation, Viral Infection and Cancer

**DOI:** 10.3389/fimmu.2021.662134

**Published:** 2021-04-19

**Authors:** Yara O. Aghabi, Alia Yasin, James I. Kennedy, Scott P. Davies, Amber E. Butler, Zania Stamataki

**Affiliations:** College of Medical and Dental Sciences, Institute for Immunology and Immunotherapy, University of Birmingham, Birmingham, United Kingdom

**Keywords:** enclysis, hepatitis, transplantation, liver autoimmunity, regulatory T cells (Treg), immune regulation, tolerance, liver cancer

## Abstract

Persistent liver inflammation can lead to cirrhosis, which associates with significant morbidity and mortality worldwide. There are no curative treatments beyond transplantation, followed by long-term immunosuppression. The global burden of end stage liver disease has been increasing and there is a shortage of donor organs, therefore new therapies are desperately needed. Harnessing the power of the immune system has shown promise in certain autoimmunity and cancer settings. In the context of the liver, regulatory T cell (Treg) therapies are in development. The hypothesis is that these specialized lymphocytes that dampen inflammation may reduce liver injury in patients with chronic, progressive diseases, and promote transplant tolerance. Various strategies including intrinsic and extracorporeal expansion of Treg cells, aim to increase their abundance to suppress immune responses. We recently discovered that hepatocytes engulf and delete Treg cells by enclysis. Herein, we propose that inhibition of enclysis may potentiate existing regulatory T cell therapeutic approaches in patients with autoimmune liver diseases and in patients receiving a transplant. Moreover, in settings where the abundance of Treg cells could hinder beneficial immunity, such us in chronic viral infection or liver cancer, enhancement of enclysis could result in transient, localized reduction of Treg cell numbers and tip the balance towards antiviral and anti-tumor immunity. We describe enclysis as is a natural process of liver immune regulation that lends itself to therapeutic targeting, particularly in combination with current Treg cell approaches.

## Introduction

The liver has a critical role in detoxification and hence it often becomes the site of cellular damage. Hepatocytes are liver epithelia that bear the worst of this process, and drive liver regeneration during injury. A healthy liver can cope with small amounts of tissue damage and repair itself as needed. Persistent liver injury, however, can lead to progressive liver damage, fibrosis, cirrhosis and end stage disease requiring a transplant.

Mortality due to liver disease is projected to overtake coronary heart disease by 2020 in the UK (1, 2). Globally, liver disease is estimated to account for 2 million deaths every year (3). Aside from preventable dietary injury, the major causes of liver failure are viral and autoimmune hepatitis. Autoimmune family liver diseases are T-cell-driven disorders that lead to hepatocyte (autoimmune hepatitis, AIH) or bile duct damage (primary biliary cholangitis, PBC, primary sclerosing cholangitis, PSC) that cause progressive liver failure. In some groups of patients that we are unable to predict, steroid or bile acid treatment respectively, can delay disease progression. Patients that fail to responsd to treatment rely on transplantation for survival. Viral hepatitis caused by hepatitis B (HBV) or hepatitis C virus (HCV), is the major indication for liver transplantation worldwide. In the UK, 180,000 patients have diagnosed chronic HBV (1) and 215,000 have persistent HCV infection (1). The WHO estimates that in 2015, HBV resulted in 887,000 deaths (4) and in 2016, HCV resulted in 339,000 deaths worldwide (5). Of these diseases that cause chronic liver inflammation, HCV is the only one where a curative treatment for the majority of patients is now available. However clinical disease progression continues in cirrhotic patients with severe disease even after virus eradication (6).

The increasing demand for liver transplantation and the decline in donor organs has highlighted the need for alternative novel therapies to prevent chronic hepatitis, which eventually leads to liver cirrhosis and increases the risk for liver cancer. ***The outcome of liver inflammation is determined by the balance of effector and regulatory immune cells activities***: chronic hepatitis arises when effector cells persist, causing liver injury. Despite their prevalence, effector cells in chronic disease fail to control hepatocellular carcinoma, one of the two cancers with mortality projected to rise by 38% by 2035 (1). There is a pressing need to discover new approaches to toggle liver inflammation to prevent liver failure (7).

The liver plays a key role in immune tolerance (8) and it hosts a rich, specialised immune compartment (9, 10). Regulatory T cells (Treg) play a critical role in dampening overactive immune responses (11, 12). Treg cells suppress immune effector function by secreting immunosuppressive cytokines, by competing with effector T cells for costimulatory molecules by depriving effector cells of IL-2 and other direct and indirect processes recently reviewed in detail by Romano and colleagues (13).

Understanding how to control Treg cell frequencies in the liver is of increasing clinical interest in transplantation, in chronic liver inflammation from multiple etiologies, and in primary and metastatic liver cancer. The clinical goal is to increase the abundance of functional Treg cells in autoimmunity (14–16) and eliminate Tregs in early infection (16, 17) in order to clear the virus and potentiate immunotherapy (12, 18).

## Enclysis in Liver Immune Regulation

**Figure 1:**
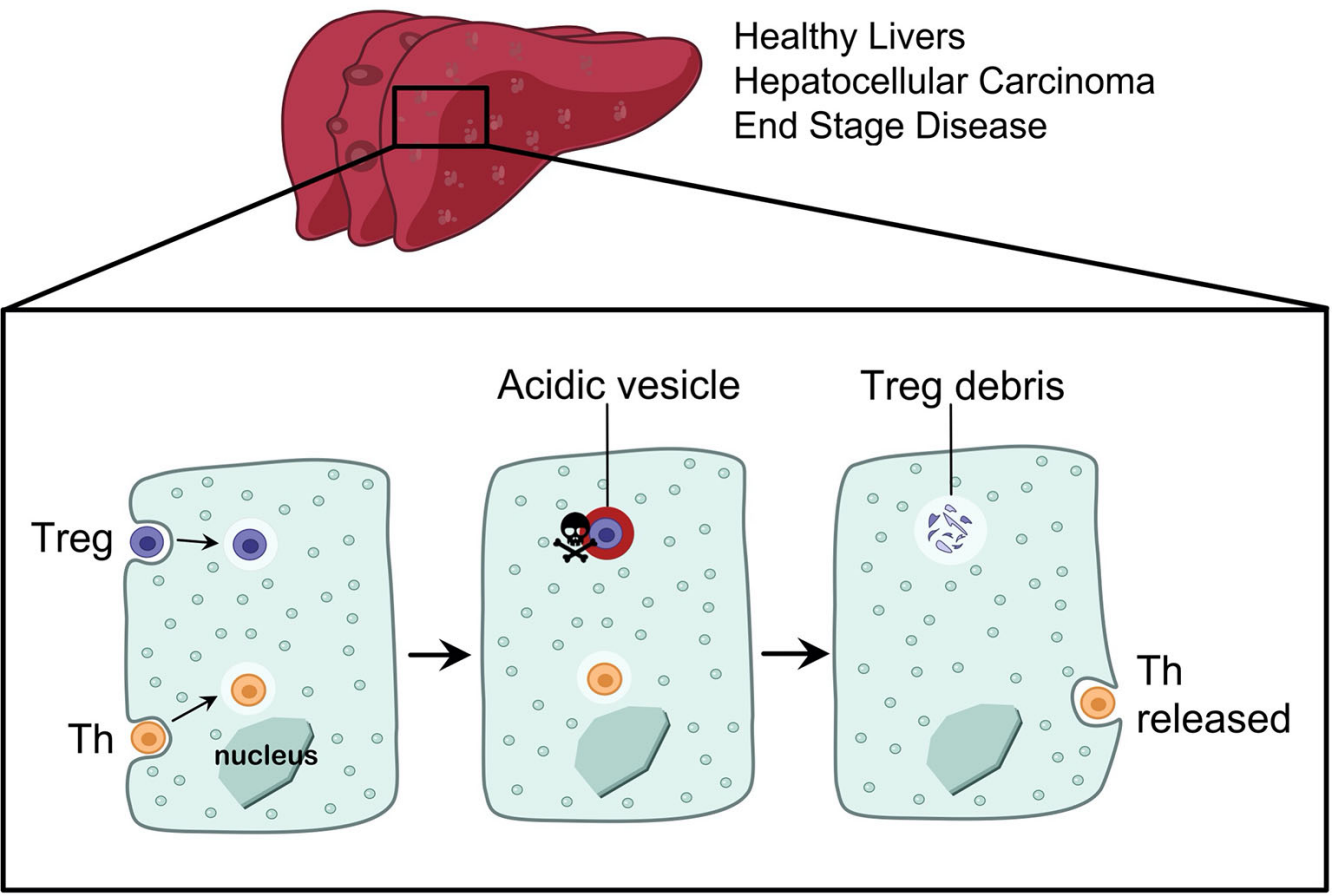
Enclysis is a CD4+ T cell engulfment process that leads to the deletion of regulatory T cells. Hepatocytes from non-cirrhotic donor livers, or from end stage disease explants, and hepatocellular carcinoma cells were all able to engulf CD4+ T cells by enclysis. T helper cells and Treg cells experience different fates inside hepatocytes, where T helper cells were preserved alive for hours or released and thus they survived enclytic capture, while Treg-containing vesicles quickly acidified.

CD4^+^ T cells are crucial cytokine-producing helper cells that orchestrate the tailoring of immune responses to fit the cause of injury. Hepatocytes comprise around 80% of the liver mass and perform vital functions including drug detoxification, clearance of dead cells (efferocytosis) and the remarkable ability of the liver to regenerate. We recently discovered that hepatocytes preferentially engulf live CD4^+^ T cells (19) in i) 2-D coculture and 3-D organoid models, ii) perfused human liver explants and iii) in healthy and end stage disease livers *in vivo*. Yet, not all T cells were treated equally: hepatocytes preferentially engulfed and deleted Treg cells, compared to effector T helper cells (Th), which promote immune responses (**Figure 1**). The incidence of Treg cells inside hepatocytes was higher in autoimmune hepatitis compared to viral hepatitis (19). We termed the engulfment and lysis of Treg cells ***Enclysis*** from the Greek *ϵγκλϵίω* (to enclose, to confine, to keep in captivity), and herein we explore how to target this process in different liver disease settings. The liver is the largest internal organ and it filters blood at a rate of ~ 1.4 L/min, enclysis could therefore have a major impact in Treg cell populations.

We demonstrated that enclysis was distinct to known cell engulfment processes (19, 20), including dead cell efferocytosis (21), the elimination of autoreactive cytotoxic T cells by suicidal emperipolesis (22) and the homotypic internalization of cancer epithelia called entosis (23, 24), it is therefore possible to target it specifically.

## Targeting Enclysis in Autoimmune Liver Disease

Loss of tolerance in the liver is observed in autoimmune family disorders such as autoimmune hepatitis (AIH), primary biliary cholangitis (PBC), and primary sclerosing cholangitis (PSC), where immune-mediated injury affects hepatocytes, small and large bile ducts respectively. The trigger for these diseases is not understood. Immunosuppression in AIH and PBC can be beneficial, however this is not the case for PSC (25). Current therapies are not curative and vary in efficacy in a manner that is presently challenging to predict for each patient, stressing the need for new treatments that effectively dampen inflammation in the liver to prevent the need for a transplant (26–29). Treg cell therapies are in various stages of development for AIH but not for cholangiopathies (25, 27). In autoimmunity, increasing the frequency of Tregs specifically in the liver by inhibiting enclysis may be useful alone or in combination with immunosuppressive regimens.

AIH is a chronic liver disease characterized by excessive immune responses associated with effector T cells (30, 31). In northern Europe, AIH affects approximately 1.9 per 100,000, with 25% of patients presenting as asymptomatic, and 40% showing signs of acute hepatitis before diagnosis (32–34). Approximately one third of patients show previous signs of increased fibrosis and liver cirrhosis (35). When treatment is readily available, a 10-year survival rate can be as high as 93%, however if no treatment is given, 40% of patients may die within six months of diagnosis (36) and current treatment shows measurable effects in up to 80% of patients (30, 37).

Various underlying liver damage mechanisms are associated with AIH. Predominantly, the presentation of self-antigens by antigen presenting cells to the T cell receptor of T-helper lymphocytes results in their activation and differentiation into more specified subsets of T-helper cells (38). For example, Th1 differentiation triggers IL-2 and interferon-gamma (IFN-γ) production, which in turn initiates activation of cytotoxic CD8^+^ T cells (39). Increased exposure of hepatocytes to IFN-γ increases the production of MHC class I complexes leading to increased T-cell activation and liver injury (13). Th2-type T cells secrete IL-4, IL-10 and IL-13, which are key cytokines for B cell maturation and the production of autoantibodies against hepatic auto-antigens, contributing further to cytotoxicity (40). Successful treatment of hepatic autoimmunity reduces hepatic inflammation and fibrosis. Our increased understanding of disease pathogenesis over the past few years suggests that a reduction in the dose and duration of steroid treatment can be considered, to minimize adverse effects associated with immunosuppression (41–43).

Evidence of Treg involvement in AIH have emerged in patients and murine models (44–46). Lapierre et al. demonstrated that severe AIH develops as a result of reduced numbers of functional Tregs, and that adoptive transfer of Tregs in mice with AIH was sufficient to restore peripheral tolerance to an ectopic liver autoantigen (FTCD), and induce remission (44). Various studies aimed to answer if Treg cell frequency or function were altered in AIH livers. Some showed numerical/functional impairment of regulatory cells in AIH (45, 47–54), and others found no deficiency in their patient cohorts (55, 56).

Although we noted no differences between FOXP3^+^ Treg cell numbers in liver tissue sections from AIH patients compared to other end stage diseases, we found that more FOXP3^+^ cells were engulfed by hepatocytes in AIH (19). Enclysis was also more frequent in FOXP3^+^ cells compared to Tbet+ helper cells, and this difference was increased in AIH patients compared to those with hepatitis B infection. Further investigation is needed to establish if the imbalance in enclysis observed with AIH would play a role in disease pathogenesis.

In patients with AIH (57, 58), as well as ten other autoimmune conditions (58), treatment with low dose IL-2 was safe, paving the way for phase II trials. Intriguingly, complexed IL-2/anti-IL-2 restored the balance between Treg and effector T cells (Teff) in the liver thereby improving the course of disease in experimental murine AIH (59). In mice with concanavalin-A-induced AIH, adoptive transfer of Tregs stimulated by allogeneic hepatic stellate cells alleviated injury (60). In CYP2D6-induced AIH mice and in AIH patients, Treg/Th17 imbalance associated with poor prognosis (61).

Supplementation with all-trans-retinoic acid (RA) or rapamycin (RP) agents enhanced Treg function and decreased up-regulation of Th1/Th2/Th17 transcription factors in cells from AIH patients (62). Further Treg conditioning experiments achieved skewing towards regulatory phenotype in cells from healthy volunteers and AIH patients, however AIH cells did not maintain suppressing function after stimulation (63). Together these data show that Treg therapies in AIH show promise, but we must carefully consider the preparation of Treg cells *ex vivo* or *in vivo*.

*Ex vivo* expansion of clinical grade regulatory T cells is laborious and costly, but it is certainly possible (64), and they can reach the liver following infusion (65). The persistence of these cells in inflamed livers and the duration of any benefit need further investigation, however in low dose IL-2-treated patients with refractory AIH, increase in Treg populations persisted until 28 days after treatment (57). Enclysis-targeting interventions could prolong efficacy of Treg therapies by preventing elimination of Treg cells in the liver parenchyma. Worryingly, immunosuppressive treatment with steroid and azathioprine diminished intrahepatic Treg cells (66). The role of immunosuppressive treatments on enclysis has not been studied. **Table 1** summarizes current and novel AIH therapies and lists the potential of enclysis inhibitors in treating AIH.

**Table 1:** AIH therapy approaches with a focus on regulatory T cells.

Type of therapy	Clinical/preclinical model	Outcomes
Corticosteroids	Immunosuppressive treatment with steroid and azathioprine diminishes intrahepatic Treg cells (66)	Life-long, does not restore liver homeostasis (25, 63)
Adoptive transfer(PolyTregs)	Xenoimmunized Type II AIH murine model (44)	Reduces the numbers of circulating autoreactive T cells and is sufficient to prevent AIH development in mice (44)
Adoptive transfer (arTregs)	Concanavalin-A-induced AIH murine model (60)	Selectively stimulates arTregs following adoptive transfer to alleviate injury and control AIH (60)
IL-2 Therapy	Murine AIH model (59) AIH patients clinical trials (57, 58)	In low dose IL-2-treated patients with refractory AIH, increases in Treg populations persisted until 28 days after treatment (57)
Retinoic acid and rapamycin agents	AIH patients clinical trial (63)	Enhances Treg function and reduces expression of Teff transcription factors (62, 63)
Enclysis Inhibitor	Enclysis inhibitors could be tested alone or in combination with existing Treg treatments	Enclysis inhibitors could potentiate Treg immunotherapy for AIH

PolyTreg, Polyclonally-expanded regulatory T cells; arTreg, Alloantigen-reactive regulatory T cells.

## Targeting Enclysis in Transplantation

As regulatory T cell therapies are in the forefront for transplantation (11, 67–69), targeting enclysis may also be useful to help prevent Graft Versus Host Disease (GVHD) and the rejection of donated organs, or to maintain remission profiles for patients post-transplant. Transplant rejection is limited solely through immunosuppressive drugs that are taken for the duration of a patient’s life, increasing risk of infection. In the liver, however, withdrawal of immunosuppression post-transplant is possible in some patients and must be managed carefully (70).

In the absence of frequent biopsies, we are unable to confirm complete absence of GVHD activity in liver patients, intervention success is therefore measured as operational tolerance. The first study to achieve operational tolerance in transplant patients was by Todo et al. where a cell product enriched for Treg cells was infused in ten patients who were weaned off immunosuppression after 18 months (71). The treatment failed to achieve operational tolerance in only three out of ten patients, all of whom suffered from autoimmune liver diseases. Furthermore, in recent phase 1 clinical trials blood Treg cells were isolated and infused into post-liver transplantation patients at 1-4.5 million/KG (68). Nine patients were administered GMP-Treg cells and it was shown that the therapy was safe and the overall Treg number increased. However, patients with autoimmune liver disease remained a challenge.

Regulatory T cell therapies are remarkably promising in transplantation and have been reviewed recently (11, 12, 72–78). To avoid broad immunosuppression and the risk of infection with polyclonal Tregs, one strategy is to focus on antigen-specific Treg cells, which induced tolerance in animal models and can also be expanded from patients (64). CAR-T cell technologies are also being explored to this end (79, 80). To aid these efforts, enclysis inhibition has the potential to prolong therapeutic Treg cell persistence in the liver by preventing their engulfment and degradation by donor liver hepatocytes. **Table 2** summarizes current approaches of regulatory T cell therapies and shows how enclysis could act in preventing liver transplant rejection and GVHD.

**Table 2:** Treg cell-focused approaches to prevent transplant rejection and graft versus host disease (GvHD).

Type of therapy	Clinical/preclinical model	Outcomes
Corticosteroids	Immunosuppression in transplant patients affects Treg number and function, reviewed in (11)	Effective in preventing rejection, associated with short- and long-term adverse events (81)
Adoptive transfer(PolyTregs)	Phase I clinical trial demonstrating safety of polyTreg therapy in addition to IL-2 therapy (68)	Operational tolerance achieved in 7/10 patients (71) Risk of infection
Adoptive transfer (arTregs)	arTregs successfully home to the liver and prevent allograft rejection in preclinical skin graft model (82)	arTregs are significantly more effective than polyTregs (64, 82)
CAR-Tregs	Tailored Treg specificity using CARs specific for antigens relevant to liver transplantation. (83–86)	Encouraging results in human and preclinical skin allograft models (87, 88)
Enclysis Inhibitor	Enclysis inhibitors should be tested alongside current immunosuppression regimens in liver transplantation	Enclysis inhibitors could potentiate immunosuppression

PolyTreg, Polyclonally-expanded regulatory T cells; arTreg, Alloantigen-reactive regulatory T cells; CAR, Chimeric Antigen Receptor.

## Targeting Enclysis in Viral Infection

Hepatitis B virus (HBV) and hepatitis C virus (HCV) are hepatotropic viruses that are the leading causes of liver transplantation worldwide. They cause progressive liver damage after decades of infection that can promote a pro-tumorigenic environment and may lead to liver failure requiring a transplant (89, 90). Chronic infection with HBV and HCV account for approximately 80% of hepatocellular carcinoma (HCC) cases worldwide (91). Every year, 1.4 million people die from viral hepatitis-related cirrhosis and liver cancer (92). An early, broad, robust T cell response has been associated with viral clearance (93, 94), and regulatory T cell frequencies are thought to hamper antiviral responses and promote persistence (90).

Sprengers et al. showed a correlation between the levels of intrahepatic CD8+ T cells and the degree of liver damage in HBV, highlighting the concept that a balance between an immune response and tolerance is essential to ensure clearance of the virus, whilst limiting any liver cell injury leading to fibrosis (95). Although Treg cells suppress immune-mediated mechanisms of liver damage, the suppression of antiviral T cell responses, which are essential for the resolution of acute HBV infection, may promote viral persistence. Indeed, vigorous cytotoxic responses are crucial to control viral infections beyond the liver (96, 97), and transient depletion of Treg cells can boost cytotoxic T cell antiviral activity (98).

There are limited data on the immunological response to acute HBV and HCV infection in the liver because of the potential complications associated with standard liver biopsies and the difficulty in detecting an often-asymptomatic acute phase. Therefore, most studies of intrahepatic Treg cells in HBV and HCV have focused on the chronic phases of infection. Elevated Treg levels in chronic HCV infection were associated with limited immunopathological damage, suggesting a critical role for Tregs in controlling the chronic inflammatory response to HCV thus limiting hepatic damage (90, 99). However, Tregs have also been shown to inhibit CD8+ T cells in chronic infection in a nonspecific manner; as they were shown to also suppress CMV-, EBV-, and HCV-specific T cells (100, 101).

Patients who spontaneously cleared HCV infection had reduced Treg cell frequencies in their blood compared to chronically infected patients after clearance, however there is a paucity of information regarding liver Treg populations in these cohorts (100–103). In chimpanzees, the sole animal model for HCV immunity, Treg cells persisted after viral clearance and aided the maintenance of HCV-specific memory T cells by regulating proliferation, *ex vivo* effector functions, and activation-induced death of HCV-specific memory T cells (104).

Another important study in this field was the low dose IL-2 treatment of ten patients with HCV-induced vasculitis (105). Extrahepatic manifestations present in some HCV-infected people and these include mixed cryoglobulinaemia with reduced peripheral blood Treg cells (106). Low dose IL-2 restored blood Treg frequencies in these patients without increasing viraemia or liver enzymes, and 8/10 showed clinical improvement (105). In this setting, increase in functional peripheral Treg numbers did not induce a viral flare; it would be interesting to investigate if hepatic Treg frequencies were affected.

In the case of HBV, it is hypothesised that persistent infection leads to transforming growth factor β (TGF-β) production from hepatic stellate cells which contributes to the differentiation of conventional CD4+ T cells into induced Treg cells (107). In chronic HCV infection, elevated serum IL-10 levels are thought to play a role in the induction of Tregs (108, 109). To address whether viral infection would offer measurable immune suppression in the context of transplantation, Bohne et al. conducted a clinical trial of immunosuppression withdrawal in 34 HCV patients, 50% of whom achieved operational tolerance. The magnitude of HCV-induced proinflammatory gene expression and the breadth of anti-HCV effector T cell responses in these patients did not influence drug withdrawal outcome (110). The authors describe an overall restrained alloreactive immune landscape in HCV patients, and viral infection did not hinder the establishment of operational tolerance.

To clear infection in patients with chronic hepatitis, reducing Treg cell frequencies in the liver in a specific and transient way may provide a sufficient boost to antiviral immunity. Increasing enclysis with enhancer compounds may reduce Treg cell numbers in the liver without depleting Tregs from the circulation. Indeed, depletion of Tregs has been shown to increase the immune control of acute HBV early in infection as the hepatitis B antigens HBeAg and HBsAg were cleared considerably faster in the serum of Treg-depleted mice compared to control. Further, early elimination of Tregs in acute HBV infection was shown to improve the recruitment of macrophages and DCs into HBV-infected livers, aiding viral clearance (16, 111). **Table 3** lists current therapies for viral hepatitis, and highlights how enclysis might be of benefit.

**Table 3:** Treatment regimens for viral hepatitis and their effects on regulatory T cells.

Therapies in viral hepatitis	Clinical model	Outcomes
Antivirals and therapeutic vaccines	Combination therapies are needed for HBV infection, including vaccines, antivirals and regimens to invigorate the liver immune response (112) HCV-targeted DAAs do not restore Treg functionality or frequency, increased after chronic infection (113)	HBV antivirals do not eliminate infection (2). Vaccine treatment reduced Treg numbers in HBV patients (114) Treg numbers could also further increase after DAA treatment for HCV (115)
IL-2 therapy	Low dose IL-2 therapy reduced peripheral blood Tregs in patients (105)	Investigation into whether intrahepatic Treg frequencies are also affected by IL-2 therapy (116)
Enclysis enhancer	Enclysis enhancement should be attempted in combination with current antiviral regimens	Increasing enclysis would reduce Treg numbers in the liver to boost antiviral immunity

DAA, Direct acting antivirals; HBV, Hepatitis B virus; HCV, hepatitis C virus.

## Targeting Enclysis in Liver Cancer

Primary liver cancer is the sixth most common type of cancer and is responsible for over 700,000 deaths annually, making it the fourth leading cause of cancer-related death worldwide (117). Hepatocellular carcinoma (HCC) is the most common malignancy in the liver, accounting for over 75% of all primary liver cancer cases (118). Malignancies in the liver, and particularly HCC, are characterized by high rates of recurrence and poor prognosis, owing mostly to the late presentation and thus late diagnosis of disease (119). As aforementioned, HBV and HCV infections are a substantial risk factor for HCC (120). Other risk factors include alcohol consumption leading to alcoholic liver disease and steatohepatitis as well as non-alcoholic fatty liver disease, associated with obesity and other metabolic disorders (121). Most HCC cases attributed to these risk factors develop in the context of cirrhosis in the liver, with up to 90% of HCC patients displaying liver cirrhosis pre-diagnosis (122).

The main treatments available for HCC, namely local ablation, surgical resection, or liver transplantation, are only viable if implemented at early stages of the disease. Thus, 80% of patients with HCC are not eligible for these treatments (123). For such patients, the first-line strategy for palliative treatment involves multi-kinase inhibitors. One such inhibitor is sorafenib, which is associated with severe adverse events (122, 124). Overall, there is a considerable unmet clinical need for patients that continue to progress or do not respond to systemic treatment. This emphasizes the need for new therapies for HCC.

It is well-established that chronic pathological inflammation is a key driver of HCC tumourigenesis in cirrhotic livers (125). Repeated and unresolved injury to hepatic tissues leads to the constitutive activation of a local immune response, resulting in the enhanced secretion of proinflammatory and mitogenic cytokines such as IL-6 and tumor necrosis factor α (TNF-α) (126). This, coupled with the subsequent recruitment of effector immune cells, results in a proinflammatory tissue microenvironment. The hypoxic environment caused by impaired blood flow to the liver further exacerbates inflammation in the cirrhotic liver. Such hostile environments drive hepatocyte apoptosis and increase the production of reactive oxygen species, driving hepatocellular mutagenesis and genomic instability, further potentiating the carcinogenic phenotype (122, 127).

The creation of a pro-inflammatory environment also stimulates the recruitment of regulatory immune cells. In HCC, tumor-infiltrating Tregs are recruited to the neoplastic site following enhanced secretion of the chemokines CCL17 and CCL2 by tumor associated macrophages (128). Once activated, these cells promote immune evasion through a variety of proposed immunosuppressive mechanisms, including the secretion of TGF-β and IL-10, the inhibition of dendritic cell activation and antigen presentation, and granzyme-dependent cytotoxicity (129). The constitutive expression of CTLA-4 is important for Treg immunosuppressive functionality, as its interaction with CD80 and CD86 ligands on the surface of dendritic cells impedes the antigen presenting cells’ activation by costimulatory molecules (130).

Different stages of HCC pathogenesis have been linked to distinct signatures of tumor-infiltrating immune cell populations (131). A large-scale analysis of tumor-infiltrating immune cells in 1090 HCC tumors revealed that greater intratumoural Treg populations were strongly correlated with poorer patient outcomes (132). This finding is consistent with several studies that also report an association between high levels of Treg infiltration and poor prognosis and response to treatment in a variety of different cancer types, including breast cancer, cervical cancer, and melanomas (133–135). In 2020, Yu et al. also reported a greater-than-3-fold increase in Treg populations in tumor tissues compared to adjacent healthy tissues, further implicating Tregs in HCC development (132). In another study, a higher proportion of Treg tumor infiltration in HCC tumors was also associated with poor tissue differentiation and advanced stages of hepatic fibrosis (136). Overall, it appears that elevated Treg numbers are associated with poor patient outcome. Thus, we propose that harnessing hepatocytes’ capacity for enclysis could provide a novel and specific mechanism of reducing Treg populations in HCC. Enhancing enclysis in the context of HCC could restrict Treg frequencies and alleviate local immunosuppression within the tumor microenvironment, aiming to reinstate tumor immunogenicity and suppress HCC tumourigenesis.

Treg cells may also augment immune dysfunction by inducing effector T cell exhaustion, characterised by inhibition of intratumoural CD8+ T cell expansion and activation (137). This exhaustive effect synergizes with neoplastic cells’ overexpression of programmed death-ligand 1 (PD-L1), activating effector T cells’ programmed cell death protein 1 (PD-1) pathway and further eliminating CD8+ T cells at tumor site (138). The various immunomodulatory mechanisms employed by Tregs contribute to the inhibition of effector immune cells in the tumor stroma, consequently depleting anti-tumor immunity and promoting immune evasion and tumor progression. Accordingly, enhanced Treg tumor infiltration was found to impede both the activation and recruitment of effector CD8+ T cells in patients with HCC (139). Tumor-infiltrating CD8+ T cells from advanced HCC tumor samples displayed significantly impaired secretion of the cytolytic enzymes perforin, granzyme A, and granzyme B. Specifically, Tregs extracted from patients with HCC were capable of significantly suppressing CD8+ T cells’ production of these cytolytic enzymes, in addition to inhibiting the secretion of the key anti-tumor cytokines IFN-γ and TNF-α *in vitro* (139). These findings support a direct role for Tregs in dampening anti-tumoral immune responses by diminishing effector T cell activation in patients with HCC.

Treg cells have been explored as a therapeutic target in a variety of different cancer types (128). These approaches mainly aim to restore and replenish intrinsic anti-tumor immune responses by eliminating the suppressive effect of Tregs within the tumor stroma. For example, in the context of melanoma, antibody blockade of CTLA-4 results in both a strong increase in both CD8+ and CD4+ T cell infiltration and a reduced proportion of intratumoural Tregs *in vivo* (140). Additionally, co-culture of CD8+ T cells extracted from HCC patients *in vitro* with Treg cells showed that Treg cells treated with anti-PD1 and anti-PD-L1 resulted in restoration of IFN-γ secretion compared to control Treg cells, which inhibited IFN-γ secretion and cytotoxicity of CD8+ T cells (141). Despite these findings, clinical trials of immune checkpoint monotherapy in patients with advanced HCC exhibited only modest improvements in patient outcomes. Treatment with the anti-CTLA-4 monoclonal antibody tremelimumab produced a less than 20% partial response rate in HCC patients (142). Furthermore, a phase III clinical study concluded that the anti PD-1 antibody nivolumab showed no superiority over sorafenib treatment in improving overall survival in patients with HCC (143), demonstrating that check point inhibition may not be sufficient to transcend liver intrinsic immune tolerance mechanisms.

Pharmacological enhancement of enclysis could be used as an adjuvant to checkpoint blockade immunotherapy, further depleting tumor-infiltrating Treg populations and thereby improving the efficacy of CTLA-4 and PD-1/PD-L1 blockade in patients with advanced HCC. As a natural liver-restricted process, targeting enclysis locally and transiently may boost anti-tumor responses in the presence or absence of check point inhibition or CAR-T cell therapy. **Table 4** lists current HCC therapies that target regulatory T cells, and highlights how enclysis enhancement might potentiate their effects to eliminate tumors in the liver.

**Table 4:** The impact of liver cancer treatments on Treg cells.

Cancer Therapies	Clinical/preclinical model	Outcomes
Multi-kinase Inhibitor (Sorafenib)	Sorafenib reduces Treg numbers in HCC (144)	Teff/Treg frequencies correlated with anti-tumor effects (144)
Immune checkpoint blockade	Immune checkpoint blockade therapy using anti-CTLA-4 reduced intratumoural Tregs *in vivo* (140) Tregs treated with anti-PD1 and anti-PD-L1 restored IFN-γ secretion in B16 melanoma (141)	Effective in B16 melanoma tumors (140). Nivolumab an anti-PD-1 antibody was no more efficacious than sorafenib treatment in improving overall survival outcome in patients with HCC (143) Treg cells may contribute to resistance to checkpoint inhibitors (145)
CAR-T cells	Tumor-targeting CAR-T cells have curative potential (146)	It is anticipated that eliminating Treg cells would be important to boost CAR-T cell therapies, particularly for solid tumors (146).
Enclysis enhancers	Enclysis enhancers could be used in combination with current immunotherapies for liver cancer.	Enclysis enhancement could potentiate antitumor immunity specifically for liver cancer (19)

HCC, Hepatocellular carcinoma; CAR, Chimeric Antigen Receptor.

## Pharmacological Interventions to Modify Enclysis

**Figure 2:**
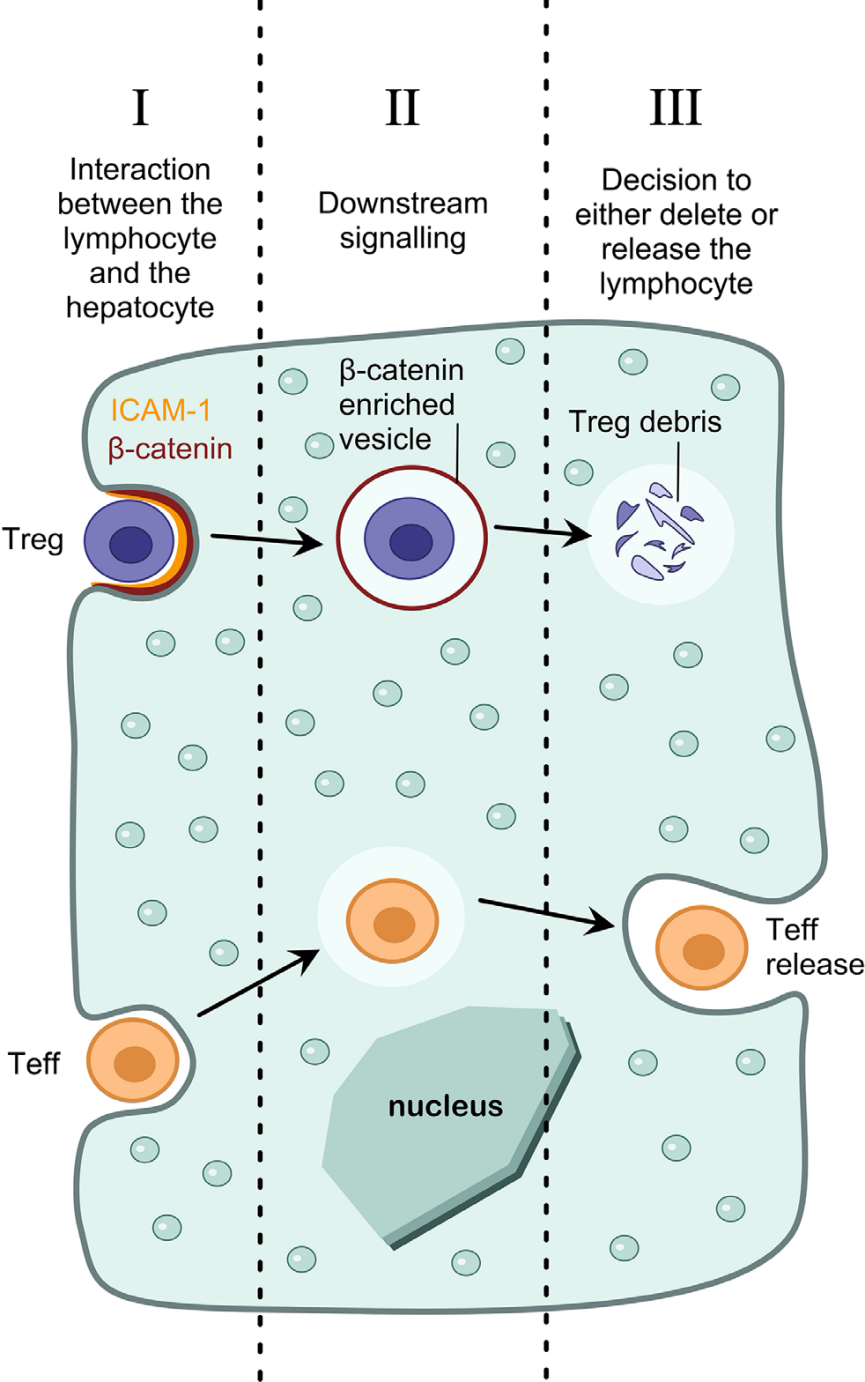
Pharmacological interventions to modulate enclysis. Understanding the mechanisms of enclysis reveals opportunities for therapeutic targeting. For example, understanding the interaction between the lymphocyte and the hepatocyte (I), the downstream signaling pathways that lead to T cell internalization (II), and the mechanism by which a hepatocyte decides the fate of the T cell (either deletion of regulatory T cells or survival and release of non-regulatory T cells) (III).

To target enclysis specifically, we must elucidate the mechanisms by which T cells are engulfed by hepatocytes, how these are specific for CD4+ T cells, and how Treg cells suffer a different fate to Teff cells (**Figure 2**). It is important to note that when considering ways to target enclysis, we must take into account housekeeping functions of hepatocytes such as phagocytosis, and prevent the modulation of these functions as a side-effect. To this end, broad endocytosis inhibitors would not be suitable to modulate enclysis.

We have shown that ICAM-1 plays a role in early adhesion of T cells to hepatocytes, and inhibiting ICAM-1 using blocking antibodies reduces enclysis (19). Despite ICAM-1 playing a role in lymphocyte adhesion, it is unlikely that it mediates enclysis specificity, since ligands for ICAM-1 (e.g. β2 integrins), are also present on other lymphocytes such as CD8+ T cells and B cells, which are not subjected to enclytic capture. Enclysis receptor interactions between T cells and hepatocytes can be inhibited using blocking antibodies, or enhanced by treatment with soluble factors that upregulate surface expression of the putative receptor(s). By targeting molecules on hepatocytes, rather than T cells, we can avoid causing broad T cell dysfunction, which would echo functionally outside the liver resulting in extra-hepatic implications.

Additionally, by understanding the signaling cascade of events after Treg cell adhesion, which result in internalization, we could target enclysis specifically. For example, we have demonstrated that enclytic vesicles are enriched in beta-catenin, and not E-cadherin, although both are important for epithelial cell-in-cell structures in cancer cells (19, 24). It would be important to understand if Wnt/β-catenin signaling pathway (147) molecules play a role in the formation of the enclytic vesicle. Cell-permeable small molecules or growth factors are ideal to modulate signaling pathways.

Finally, we have demonstrated that hepatocytes are able to make a distinction between regulatory T cells (which results in their degradation) and helper T cells (which results in survival and release) (19). Understanding the mechanisms by which hepatocytes make this distinction will reveal new molecular targets for therapeutic intervention. For example, to increase the frequency of regulatory T cells in the liver, we could manipulate the hepatocyte such that it blocks Treg cell degradation in enclytic vesicles, and rather directs the cells towards a pathway reserved for the release of T helper cells.

## Future Perspectives and Concluding Remarks

As Mujal and Krummel elegantly discussed (148), it is simplistic to consider immunity as an on/off switch between activation and tolerance. The outcome of complex immune responses is best described as a continuum, where synergistic effects from multiple cell types and processes protect from autoimmunity while permitting efficient viral clearance and tumor elimination. Regulatory T cell activity is part of this continuum, and we are beginning to understand how these cells can be employed most effectively in the tolerising microenvironment of the liver.

In viral infection, the liver needs to deal with abundant Treg populations swiftly to mount an effective immune response, thus Tregs cross the sinusoidal endothelial layers into the parenchyma. Hepatocytes engulf them by enclysis to rapidly tip the balance towards inflammation when needed. At the resolution of infection, the endothelial barriers are restored and Treg populations increase again to restore tolerance.

**Figure 3:**
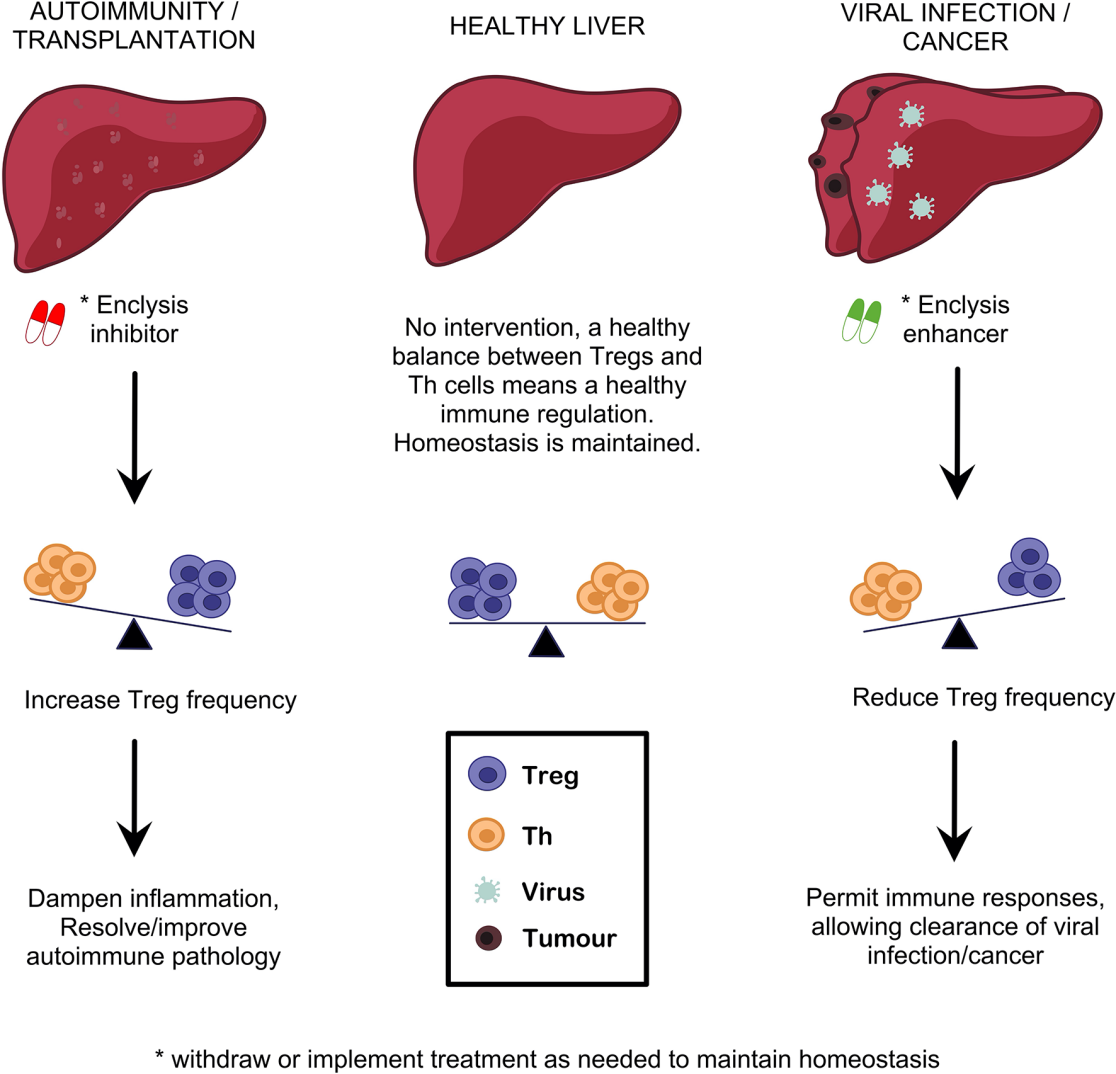
Targeting enclysis to restore immune regulation in autoimmune liver disease, transplantation, viral infection and liver cancer. Enclysis is a natural process with the potential to alter regulatory T cell frequencies specifically in the liver, and potentiate existing Treg therapies. We propose that enclysis inhibition may augment Treg abundance where most needed, to dampen overactive immune responses in autoimmunity or transplantation. Conversely, enclysis-enhancing compounds would aid local, transient elimination of Treg cells to jump-start exhausted antiviral or anti-tumor responses.

We described how inhibition of enclysis could increase frequencies of Tregs in AIH and in transplantation, where regulatory cell-targeting approaches have started to yield promising results. Conversely, we propose use of enclysis-enhancing drugs to promote Treg deletion in a well-controlled transient and liver-specific setting, to boost antiviral and anti-tumor responses where necessary (**Figure 3**). Small interventions such as enclysis modulation may be sufficient to restore healthy immune regulation in livers where chronic inflammation has caused immune dysfunction. This fits well with the “accommodation archetypes” described by Mujal and Krummel (148).

## Author Contributions

YA, AY, JK, AB and ZS researched and composed the review. YA prepared the figures. SD provided helpful critique of the manuscript. YA and ZS finalized the review. All authors contributed to the article and approved the submitted version.

## Funding

This work is supported by an MIBTP studentship to YA (BBSRC, UKRI, grant number BB/T00746X/1), an NC3R trainee postdoctoral fellowship to SD (UKRI) and an MRF intermediate career fellowship to ZS (UKRI, grant number NC/R002061/1) and an MRF intermediate career fellowship to ZS (UKRI, grant number MRF-169-0001-F-STAM-C0826).

## Conflict of Interest

The authors declare that the research was conducted in the absence of any commercial or financial relationships that could be construed as a potential conflict of interest.
